# *IL1A* regulates MSU-induced apoptosis and inflammatory response through TLR4/MyD88/NF-κB signaling pathway

**DOI:** 10.7150/ijms.112102

**Published:** 2025-06-23

**Authors:** Fei Pan, Yun Zhang, Min Li, Mei Liu

**Affiliations:** 1 Department of General Medicine, Minhang Hospital, Fudan University, 170 Xinsong Road, 201199, Shanghai, China.; 2 Teaching Management Section, Xinzhuang Community Health Service Center, 1099 Shuiqing Road, Minhang District, 201199, Shanghai, China.

**Keywords:** *IL1A*, Apoptosis, Inflammatory, Gout, TLR4/MyD88/NF-κB signaling pathway

## Abstract

**Background:** The increased inflammation associated with interleukin-1α (*IL-1A*) induced by monosodium urate (MSU) crystal-induced gouty arthritis is mediated through the NLRP3 inflammasome and NF-κB signaling pathways. This study investigated the role of *IL1A* in MSU-mediated inflammation and its therapeutic potential.

**Methods:** MSU crystals were applied to THP-1 macrophages and human vascular endothelial cells (HUVECs) with or without *IL1A* knockdown. Quantitative reverse transcription polymerase chain reaction (qRT-PCR), western blotting (WB), cell counting kit-8 (CCK-8), flow cytometry, and enzyme-linked immunosorbent assay (ELISA) were used to assess *IL1A* expression, cell viability, apoptosis, and the generation of inflammatory cytokines. The activation of the NLRP3 inflammasome and TLR4/MyD88/NF-κB pathways was evaluated, with TAK-242 used to assess synergistic effects.

**Results:** MSU increased *IL1A* expression, induced apoptosis, and reduced cell viability in HUVECs, effects reversed by* IL1A* knockdown. *IL1A* knockdown media mitigated apoptosis in HUVECs exposed to conditioned media from MSU-stimulated THP-1 macrophages. *IL1A* knockdown reduced MSU-induced proinflammatory cytokines and NLRP3 activation in THP-1 cells. TAK-242 showed synergistic effects, while *IL1A* knockdown inhibited TLR4/MyD88/NF-κB activation.

**Conclusions:** *IL1A* promotes cell death and inflammation in MSU-induced gout. Knockdown of *IL1A* mitigates these effects, suggesting it may be a potential therapeutic target for MSU-induced inflammation.

## 1. Introduction

Gout is a common inflammatory arthritis characterized by the deposition of monosodium urate (MSU) crystals within joints, leading to sudden and excruciating episodes of pain, swelling, and redness^1^, ^2^. The crystallization of uric acid into MSU is primarily driven by hyperuricemia, often associated with genetic predisposition, obesity, excessive alcohol intake, and the use of certain medications that alter uric acid metabolism^3^, ^4^. Diagnosing acute gouty arthritis relies on clinical symptoms, polarized light microscopy, and synovial fluid analysis to detect birefringent MSU crystals^5^. The pathogenesis of gout involves a complex interplay between MSU crystals and the innate immune system. MSU crystals are recognized as danger-associated molecular patterns (DAMPs), activating immune cells through pattern recognition receptors such as the Toll-like receptor (TLR) family, particularly TLR2 and TLR4^6^-^8^. This recognition initiates downstream signaling cascades, including activation of the NF-κB transcription factor and assembly of the NLRP3 inflammasome complex^9^, ^10^. The activation of NF-κB leads to the transcription of pro-inflammatory cytokines such as tumor necrosis factor-α (TNF-α) and interleukin-1β (IL-1β), while the NLRP3 inflammasome promotes maturation and secretion of IL-1β, amplifying the inflammatory response^11^, ^12^.

Recent studies have demonstrated that pharmacological agents targeting these pathways, such as palmatine and Phellinus igniarius polyphenols, can effectively reduce MSU-induced oxidative stress and inflammation by modulating the TLR4/NF-κB/NLRP3 signaling pathway^13^, ^14^. These findings underscore the critical role of innate immune signaling pathways in the progression of gout and suggest promising therapeutic avenues.

Although IL-1β has been extensively studied in the pathophysiology of gout, the role of IL-1α remains relatively underexplored. IL-1α, produced by activated macrophages, epithelial cells, and other immune cells, acts as an early alarm cytokine in sterile inflammatory responses^15^, ^16^. It can initiate inflammatory signaling independently of inflammasome activation by binding to the IL-1 receptor and activating downstream NF-κB and MAPK pathways^17^. Dysregulated IL-1α expression has been implicated in the pathogenesis of chronic inflammatory conditions, such as rheumatoid arthritis and inflammatory bowel disease 18-20. Moreover, evidence suggests that IL-1α may enhance NLRP3 inflammasome activity, contributing to a feed-forward loop that amplifies inflammation^21^.

Given that MSU crystals activate both TLR and inflammasome pathways, and considering the pro-inflammatory role of IL-1α in related diseases, it is plausible that IL-1α may play a significant yet overlooked role in MSU-induced gouty inflammation. However, the specific contribution of IL-1α to MSU-triggered cellular apoptosis, inflammatory cytokine production, and pathway activation remains unclear. Therefore, the present study aims to investigate the functional role of IL-1α (*IL1A*) in MSU-induced inflammatory responses and apoptosis. We hypothesize that *IL1A* knockdown could attenuate MSU-mediated activation of the TLR4/MyD88/NF-κB signaling axis and the NLRP3 inflammasome, thereby reducing the production of pro-inflammatory cytokines and protecting endothelial and macrophage cells from inflammation-induced injury.

## 2. Materials and Methods

Human umbilical vein endothelial cells (HUVEC) and human mononuclear leukemia cell line (THP-1) were used in this study to mimic the inflammatory microenvironment of gouty arthritis. HUVEC, as an endothelial cell model, helps to study the role of vascular endothelial cells in the inflammatory response induced by MSU crystals^7^, ^22^. THP-1 cells, as a macrophage model, help to study the role of macrophages in the inflammatory cascade response^23^, ^24^. By co-culturing these two cell types, we aimed to mimic the complex inflammatory cell interactions in gout patients and thus more accurately investigate the mechanism of *IL1A*'s role in MSU-induced inflammatory responses^15^, ^25^.

### 2.1 Cell lines and culture

The Chinese Academy of Science Cell Bank in Shanghai, China, provided human monocytic leukemia (THP-1) and human umbilical vein endothelial cells (HUVEC). Cells were cultured at low passage numbers (passage number < 10) to maintain their original characteristics. All cells were tested and confirmed to be mycoplasma-free before use Supplementary Figure 1. HUVECs were cultured at 37°C in a humidified environment with 5% CO_2_ in Dulbecco's Modified Eagle Medium (DMEM) supplemented with 10% fetal bovine serum (FBS) (Gibco), 100 U/mL penicillin, and 100 µg/mL streptomycin. Under the same conditions, THP-1 cells were cultivated in RPMI 1640 medium (Gibco) supplemented with 10% FBS, 100 U/mL penicillin, and 100 µg/mL streptomycin.

### 2.2 Cell Treatment

For 6 hours, HUVEC cells were treated with 50 μg/mL and 100 μg/mL of MSU (Sigma-Aldrich, USA). THP-1 cells were treated with 250 ng/mL of phorbol myristate acetate (PMA) for 3 h to differentiate into macrophages. Differentiated THP-1 macrophages are cultured overnight with fresh Opti-MEM medium. THP-1 macrophages were stimulated with 100 μg/mL MSU crystals for 24 hours. After stimulation, the supernatant, which is conditioned medium 26, is collected. Centrifuge the CM (3,000 rpm, 10 min) to remove cell debris and undissolved MSU crystals. Collected CMs are stored at -80 °C for subsequent experiments. TAK-242 (1 μM), an inhibitor of TLR4, was applied for 24 hours to treat THP-1 cells.

### 2.3 Treatment of HUVECs with CM

HUVECs are seeded in 96-well plates at a density of 5 × 10^3^ cells/well and cultured overnight to allow them to adhere to the wall. HUVECs are then treated with the CM prepared above for 6 hours to assess its effect on the cells. CM was not diluted, and full-strength CM was used. The control group is treated with CM from untreated THP-1 macrophages.

### 2.4 Cell transfection

HUVECs and THP-1 cells were arranged in 24-well plates at a density of 2 × 10^5^ cells per well for transient transfection. To specifically reduce *IL1A* expression, we transfected cells (si-*IL1A*) with a small interfering RNA (siRNA) specific for *IL1A* mRNA with the following sequence: UAGCAACCAACGGGAAGGUUCUGAA (Sense Sequence). UUCAGAACCUUCCCGUUGGUUGCUA (Antisense Sequence). As a control, another group of cells was transfected with non-specific siRNA (si-NC) that did not target any intracellular mRNA, with the following sequences: UAGACCAGGCAGAAGCUUGUACGAA (Sense Sequence); UUCGUACAAGCUUCUGCCUGGUCUA (Antisense Sequence). These siRNA products were synthesized by GenePharma (Shanghai). As directed by the manufacturer, Lipofectamine 3000 (Invitrogen, USA) was used to transfect cells. After transfection, cells were cultured at 37°C with 5% CO_2_ for 24 hours to ensure efficient siRNA introduction and knockdown effect. 24 hours later, cells were harvested for subsequent processing and analysis.

### 2.5 Quantitative real-time polymerase chain reaction (qRT-PCR)

The total RNA used in the experiment was processed according to the general qRT-PCR experimental method^27^. Levels of gene expression were measured and adjusted for GAPDH. The 2^-ΔΔCT^ method was employed to compute each target expression level. Table 1 contains primer sequences.

### 2.6 Western blot (WB) assay

RIPA lysis buffer with phosphatase and protease inhibitors (Beyotime, Shanghai, China; Applygen, Beijing, China) was used to obtain protein lysates from HUVEC and THP-1 cells. The protein concentration was established using the BCA Protein Assay Kit (Beyotime, China). 10% SDS-PAGE was used to separate equal quantities of protein, which were then transferred to PVDF membranes (Beyotime, Beijing, China). After 5% skim milk was used to block the membranes, primary (IL-1α, ab9614, 1:1000, Bax, ab32503, 1:1000, Bcl-2, ab1828581, 1:2000, Cleaved Caspase3, ab32351, 1:5000, MyD88, ab133739, 1:1000, NLRP3, ab263899, 1:1000, ASC, ab155970, 1:1000, CASP1, ab207802, 1:1000, p-NF-κB, ab76302, 1:1000, NF-κB, ab32536, 1:1000, Abcam, China; p-IκBα, Cat No. 82349-1-RR, 1:5000, IκBα, Cat No. 10268-1-AP, 1:5000, TLR4, Cat No. 19811-1-AP, 1:1000, Wuhan Sanying, China) and suitable secondary antibodies were added and incubated. GAPDH (ab181602, Abcam, China, 1:10000) was an internal reference. The protein bands were seen using an enhanced chemiluminescence (ECL) kit (Tiangen, Beijing, China), and the pictures were recorded using a ChemiDoc imaging system (Bio-Rad, Shanghai, China). WB band intensity is quantified using ImageJ software. GAPDH is used as an internal reference protein to reflect the relative expression levels of the target protein.

### 2.7 Cell Counting Kit-8 (CCK-8) assay

The Cell Counting Kit-8 (CCK-8) test (KeyGEN, Nanjing, China) was employed to assess the viability of HUVEC cells. After being seeded in 96-well plates at a density of 5 × 10³ cells per well, the cells were allowed to adhere overnight before treatment. Following the treatments, 10 µL of CCK-8 reagent was added to each well. The cells were then incubated with the reagent at 37°C for 2 hours. The absorbance at 450 nm was measured using a Thermo Fisher Scientific microplate reader to assess cell viability.

### 2.8 Flow cytometry

HUVECs were harvested using trypsin-EDTA (Life Technologies Inc., Beijing, China) and then washed with phosphate-buffered saline (PBS) to prepare for flow cytometry analysis. Staining was performed using Annexin V-FITC and propidium iodide (PI) according to the manufacturer's protocol to distinguish between live, apoptotic, and necrotic cells. The stained cells were analyzed using a flow cytometer (Jiyuan, Guangzhou, China), and the data were processed to calculate the cell apoptosis rate using FlowJo software (FlowJo, Hangzhou, China).

### 2.9 Enzyme-linked immunosorbent assay (ELISA)

An ELISA technique was used to determine the TNF-α, iNOS, IL-1β, and IL-6 in the supernatants of cell cultures. Cell culture supernatants were collected and suitably diluted before being added to an ELISA plate previously coated with antibodies specific to the inflammatory factors. Following many washing steps and incubation, an enzyme-linked secondary antibody was added, and a chromogenic substrate was used to produce the reaction. After stopping the process, a microplate reader measured the absorbance at the required wavelength. A standard curve was developed using known amounts of each target protein to calculate the quantities of inflammatory factors. The results were expressed as protein pg/mg.

### 2.10 Luciferase reporter gene assay

To measure the transcriptional activity of NF-κB in THP-1 cells, NF-κB luciferase reporter assays were employed Observing the guidelines provided by the manufacturer, a reporter plasmid for NF-κB luciferase (NF-κB-Luc, Genomeditech, Shanghai, China) was used to transfect cells using Lipofectamine^TM^ 3000 (ThermoFisher Scientific, Shanghai, China). Following treatments, the Dual-Luciferase® Reporter Assay System and a microplate luminometer (Promega, E1910) were used to measure luciferase activity. To measure relative NF-κB activation, firefly luciferase activity was adjusted to Renilla luciferase activity as an internal reference.

### 2.11 Statistical analysis

The R software was used for statistical analysis. Each experiment was conducted with three independent biological replicates, and the mean ± standard deviation (SD) was used to describe the results. For post-hoc analysis, Tukey's test was employed, and one-way ANOVA was employed to ascertain if the differences were significant. The criterion for statistical significance was set at *P* < 0.05.

## 3. Results

### 3.1 MSU-induced IL1A upregulation in HUVECs and the effect of IL1A knockdown

**Figure 1:**
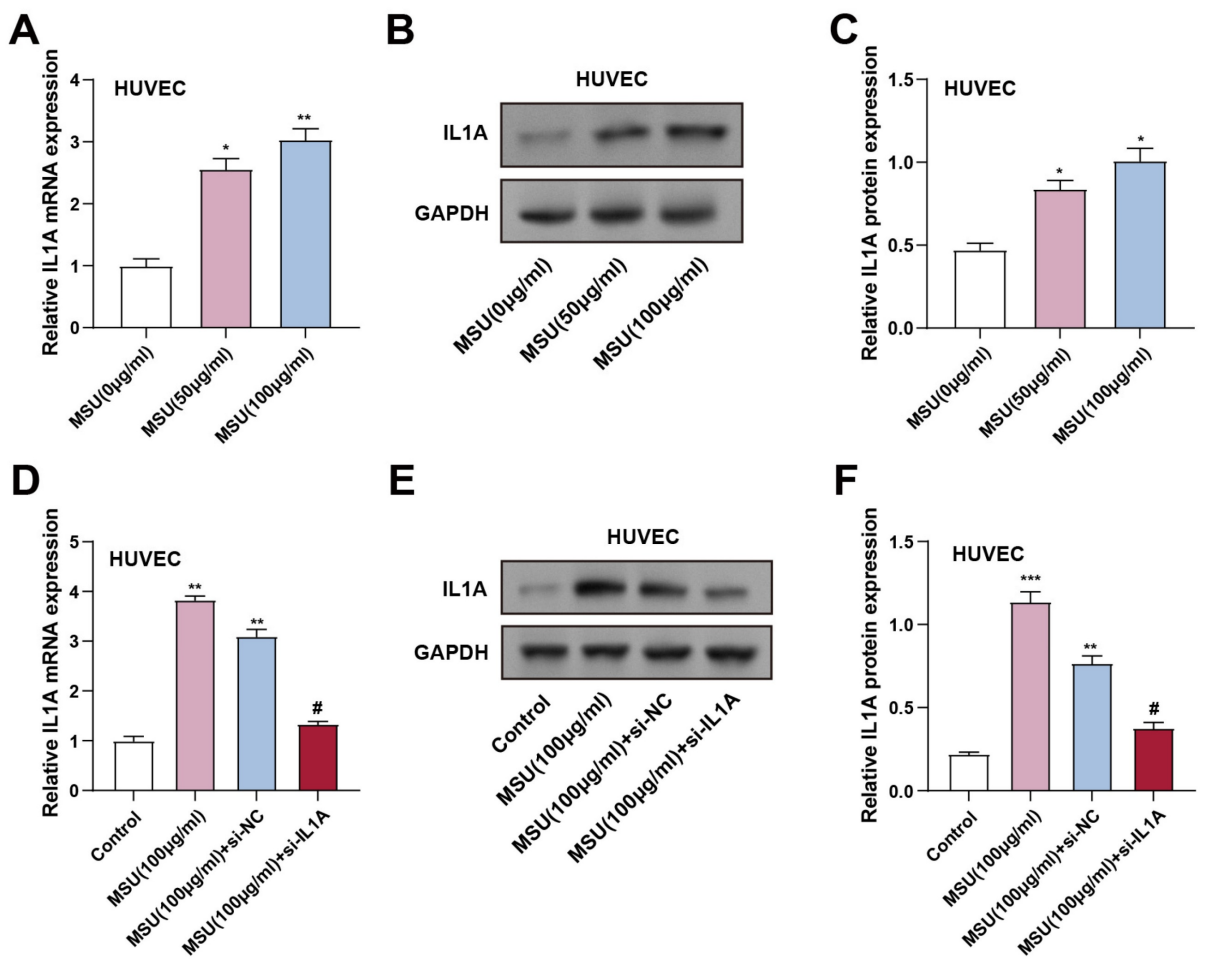
***IL1A* expression increased in HUVECs after MSU treatment.** (A) qRT-PCR was performed to detect the expression of *IL1A* in HUVECs treated under different conditions. ^*^P < 0.05, ^**^P < 0.01 vs. MUS (0 μg/ml). (B and C) WB images of IL1A expression after treatment with different concentrations of MSU (B) and quantification of IL1A protein expression (C). *P < 0.05 vs. MUS (0 μg/ml). (D) qRT-PCR detection of *IL1A* mRNA expression changes in HUVECs under different treatment conditions. ^**^P<0.01 vs. Control; ^#^*P*<0.05 vs. MSU (100 μg/ml) + si-NC. (E and F) WB detection of IL1A protein expression changes in HUVECs under different treatment conditions and quantification results. ^**^P<0.01, ^***^P<0.001 vs. Control; ^#^*P*<0.05 vs. MSU (100 μg/ml) + si-NC. qRT-PCR, quantitative real-time polymerase chain reaction; NC, negative control; MSU, monosodium urate; HUVECs, Human Umbilical Vein Endothelial Cells.

The expression levels of *IL1A* gene and IL-1α protein in MSU-treated HUVECs were assessed using qRT-PCR and WB (0 μg/mL for control and 50 and 100 μg/mL for experimental groups). HUVECs were co-incubated with MSU for 3 hours. The results showed that *IL1A* gene and IL-1α protein expression significantly increased in cells treated with 50 and 100 μg/mL MSU compared to the control group (Figures 1A-1C). To confirm the effectiveness of *IL1A* knockdown, HUVECs were transfected with si-*IL1A*. qRT-PCR and WB analyses confirmed that *IL1A* gene and IL-1α protein expression were significantly up-regulated in 100 μg/mL MSU-treated HUVECs compared with controls. However, co-treatment with si-*IL1A* in MSU-treated cells effectively suppressed this up-regulation, suggesting that siRNA-mediated knockdown of *IL1A* attenuated the MSU-induced increase in *IL1A* gene and IL-1α protein expression (Figures 1D-1F).

### 3.2 Downregulation of IL1A inhibits the apoptotic effect of MSU on HUVECs

**Figure 2:**
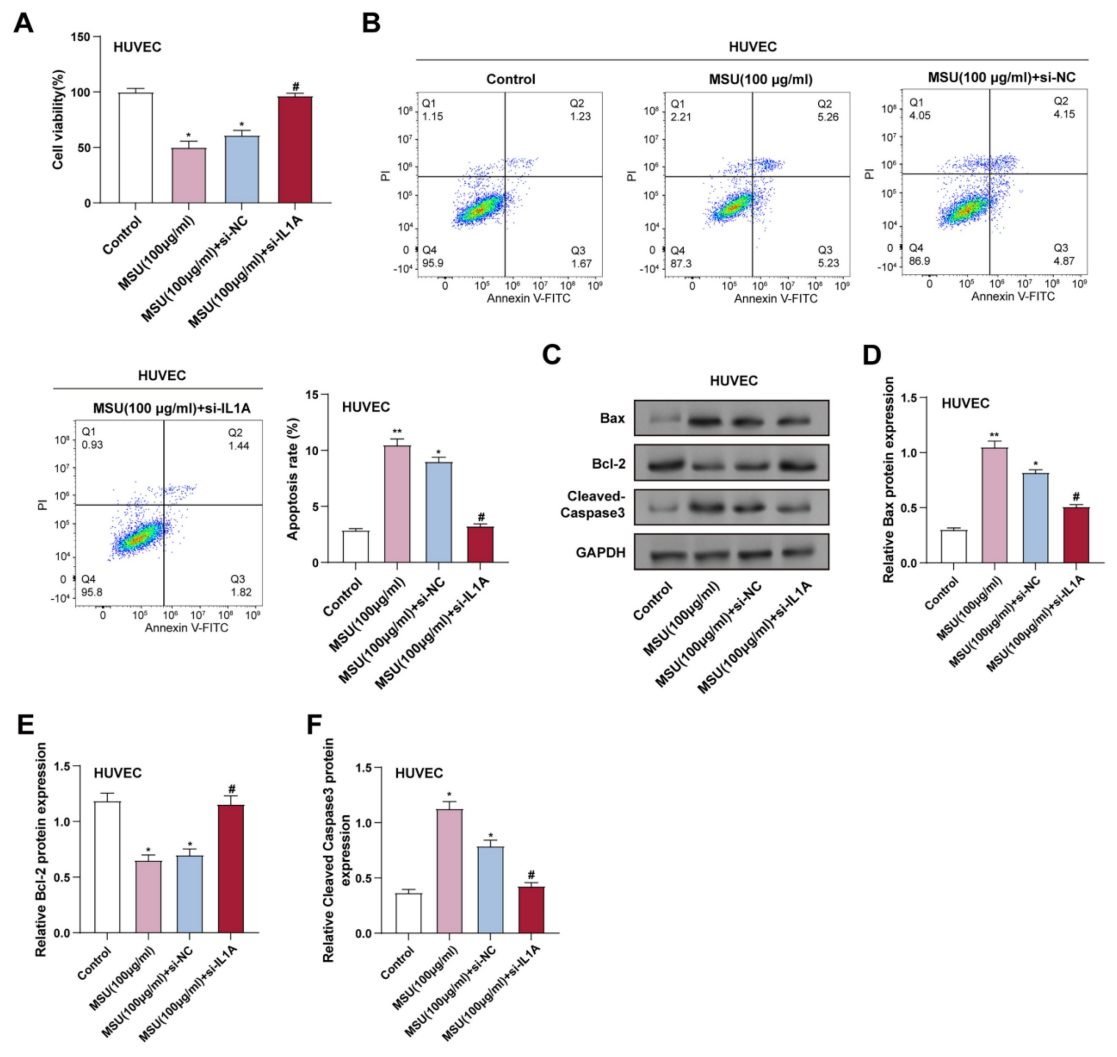
** Downregulation of *IL1A* inhibits the apoptotic effect of MSU on HUVECs.** (A) CCK-8 assay was used to measure the cell viability of HUVECs under different conditions. ^*^P<0.05 vs. Control; ^#^*P*<0.05 vs. MSU (100 μg/ml) + si-NC. (B) Representative flow cytometry graphs illustrating the apoptosis of HUVECs treated under various conditions. ^*^P<0.05, ^**^P<0.01 vs. Control; ^#^*P*<0.05 vs. MSU (100 μg/ml) + si-NC. (C-F) WB analysis of the expression of apoptosis-related proteins Bax, Bcl-2, and Cleaved Caspase-3 in HUVECs under different conditions. Data were normalized to GAPDH as an internal control. ^*^P<0.05, ^**^P<0.01 vs. Control; ^#^*P*<0.05 vs. MSU (100 μg/ml) + si-NC. CCK-8, Cell Counting Kit-8; WB, Western blot; NC, negative control; MSU, monosodium urate; HUVEC, Human Umbilical Vein Endothelial Cells.

CCK-8 assay showed that treatment with 100 μg/ml MSU significantly induced cell death in HUVECs after 3 hours of incubation, and *IL1A* knockdown effectively reversed this effect (Figure 2A). Consistently, flow cytometry results showed a significant increase in apoptosis after 3 hours of MSU treatment, which was attenuated by *IL1A* knockdown (Figure 2B). Apoptosis was assessed in flow cytometry experiments using Annexin V-FITC and PI as markers. Annexin V-FITC-positive and PI-negative cells were considered early apoptotic cells, whereas Annexin V-FITC- and PI-positive cells were considered late apoptotic or necrotic cells. WB analysis further assessed the expression of proteins associated with apoptosis. 3 hours of MSU treatment resulted in a significant upregulation of Bax and cleaved Caspase-3, and *IL1A* knockdown attenuated these effects. In contrast, MSU significantly reduced Bcl-2 expression after 3 hours of treatment, which was attenuated by *IL1A* knockdown (Figures 2C-2F).

### 3.3 MSU-induced IL1A upregulation in THP-1 macrophages and the impact of IL1A knockdown

**Figure 3:**
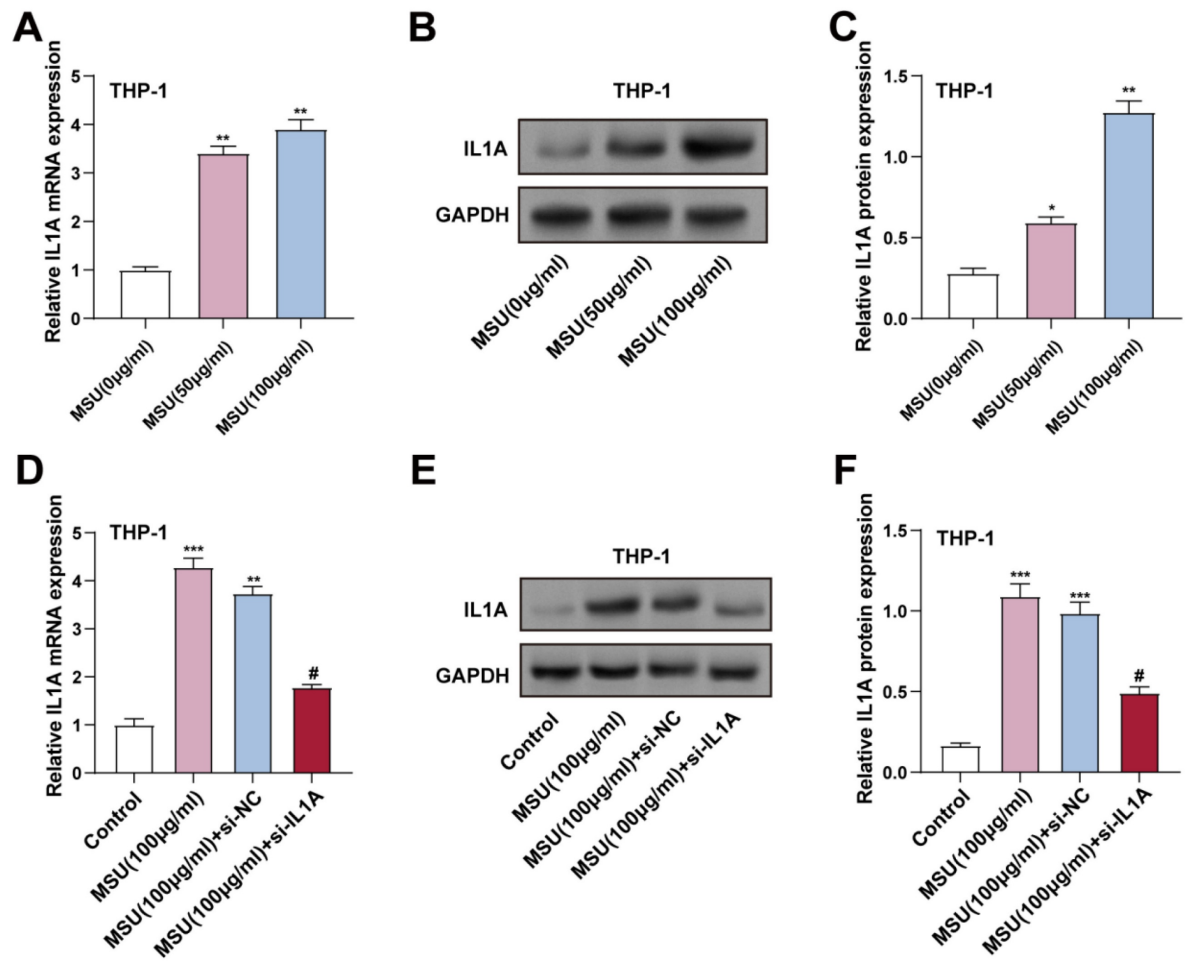
***IL1A* expression increased in THP-1 cells after MSU treatment.** (A) qRT-PCR was performed to detect the expression of *IL1A* in THP-1 cells treated under different conditions. ^**^P < 0.01 vs. MUS (0 μg/ml). (B and C) WB analysis of protein *IL1A* expression in THP-1 cells treated under different conditions. ^*^P < 0.05, ^**^P < 0.01 vs. MUS (0 μg/ml). (D) qRT-PCR was performed to analyze the changes in *IL1A* mRNA expression in THP-1 cells treated under different conditions. ^**^P < 0.01, ^***^P < 0.001 vs. Control; ^#^*P* < 0.05 vs. MSU (100 μg/ml) + si-NC. (E and F) WB analysis of *IL1A* protein expression in THP-1 cells treated under different conditions. ^***^P < 0.001 vs. Control; ^#^*P* < 0.05 vs. MSU (100 μg/ml) + si-NC. qRT-PCR, quantitative real-time polymerase chain reaction; NC, negative control; MSU, monosodium urate.

To investigate how MSU affects the production of *IL1A* gene and IL-1α protein in THP-1 cells, we treated these cells with 0 (control), 50, and 100 μg/mL levels of MSU for 24 hours. We assessed the expression levels of the *IL1A* gene and IL-1α protein by qRT-PCR and WB. These findings indicated that *IL1A* gene and IL-1α protein expression were significantly higher in cells administered with 50 and 100 μg/mL MSU compared to the control group (Figures 3A-3C). Notably, 100 μg/mL MSU caused a higher *IL1A* upregulation than 50 μg/mL, indicating a dose-dependent effect. To confirm the efficiency of *IL1A* knockdown, THP-1 cells were transfected with si-*IL1A*. qRT-PCR and WB confirmed that *IL1A* gene and IL-1α protein expression were significantly regulated by MSU treatment combined with si-*IL1A* intervention. Specifically, 100 μg/mL MSU treatment significantly up-regulated *IL1A* gene and IL-1α protein expression, whereas co-treatment with si-*IL1A* significantly attenuated this up-regulation in MSU-treated cells, effectively decreasing the levels of *IL1A* gene and IL-1α protein in THP-1 macrophages compared with those in the group receiving MSU alone (Figures 3D-3F).

### 3.4 IL1A downregulation inhibits MSU-CM-induced HUVECs apoptosis

**Figure 4:**
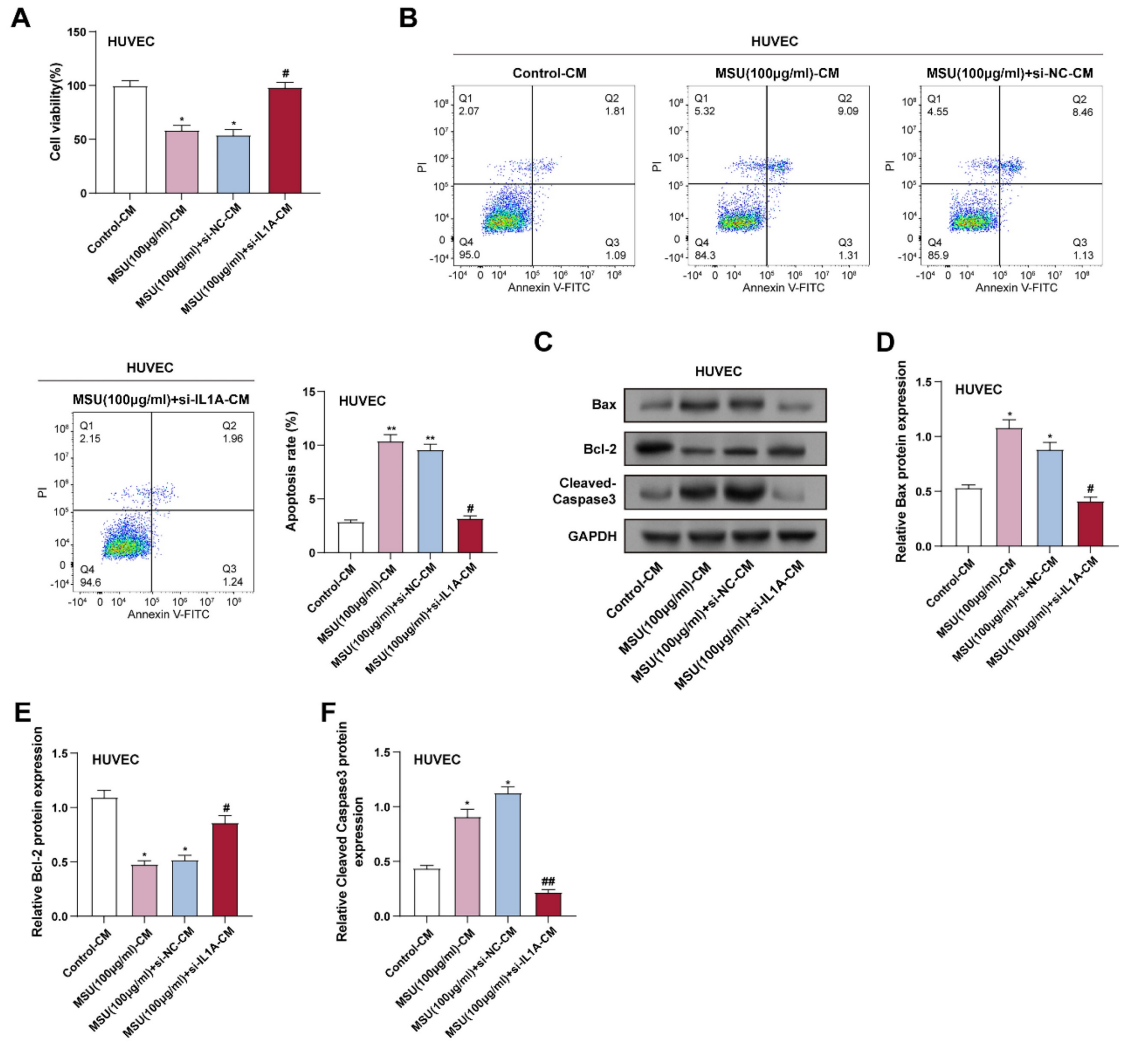
***IL1A* downregulation inhibits MSU-CM-induced HUVECs apoptosis.** (A) CCK-8 analysis of the effect of macrophage-conditioned medium obtained by treatment under different conditions on HUVEC cell proliferation. ^*^P < 0.05 vs. Control; ^#^*P* < 0.05 vs. MSU (100 μg/ml) + si-NC-CM. (B) Flow cytometry analysis of the effect of macrophage-conditioned medium obtained from cells treated under different conditions on the apoptotic rate of HUVEC cells. ^**^P < 0.01 vs. Control; ^#^*P* < 0.05 vs. MSU (100 μg/ml) + si-NC-CM. (C) WB analysis of protein expression of apoptosis-related proteins Bax, Bcl-2, and Cleaved Caspase-3 in HUVEC cells treated with macrophage-conditioned medium under different conditions. (D-F) Quantification of Bax (D), Bcl-2 (E), and Cleaved Caspase-3 (F) protein levels. ^*^P < 0.05 vs. Control; ^#^*P* < 0.05, ^##^*P* < 0.01 vs. MSU (100 μg/ml) + si-NC-CM. CCK-8, Cell Counting Kit-8; NC, negative control; CM, conditioned medium; MSU, monosodium urate; HUVECs, Human Umbilical Vein Endothelial Cells.

CM was collected from THP-1 macrophages given 100 µg/mL of MSU to create an inflammatory environment. CCK-8 assay showed that CM from MSU-treated macrophages (MSU-CM) significantly inhibited HUVECs' viability. In contrast, CM from THP-1 macrophages treated with both MSU and si-*IL1A* (MSU + si-*IL1A*-CM) significantly improved HUVECs' viability compared to MSU-CM alone (Figure 4A). Flow cytometry analysis further confirmed that MSU + si-*IL1A*-CM reduced the apoptosis rate of HUVECs induced by MSU-CM (Figure 4B). The expression of proteins linked to apoptosis in HUVECs was assessed by WB analysis and compared with treatment with MSU-CM alone; treatment with MSU + si-*IL1A*-CM resulted in a decrease in the pro-apoptotic protein (Cleaved caspase-3) and an increase in the anti-apoptotic protein (Bcl-2) (Figures 4C-4F). These results indicated that downregulation of *IL1A* in HUVECs attenuated the pro-apoptotic effect of MSU-CM on HUVECs, suggesting its potential protective effect against inflammation-induced cellular stress.

### 3.5 IL1A knockdown attenuates MSU-induced activation of the TLR4/MyD88/NF-κB signaling pathway in macrophages

**Figure 5:**
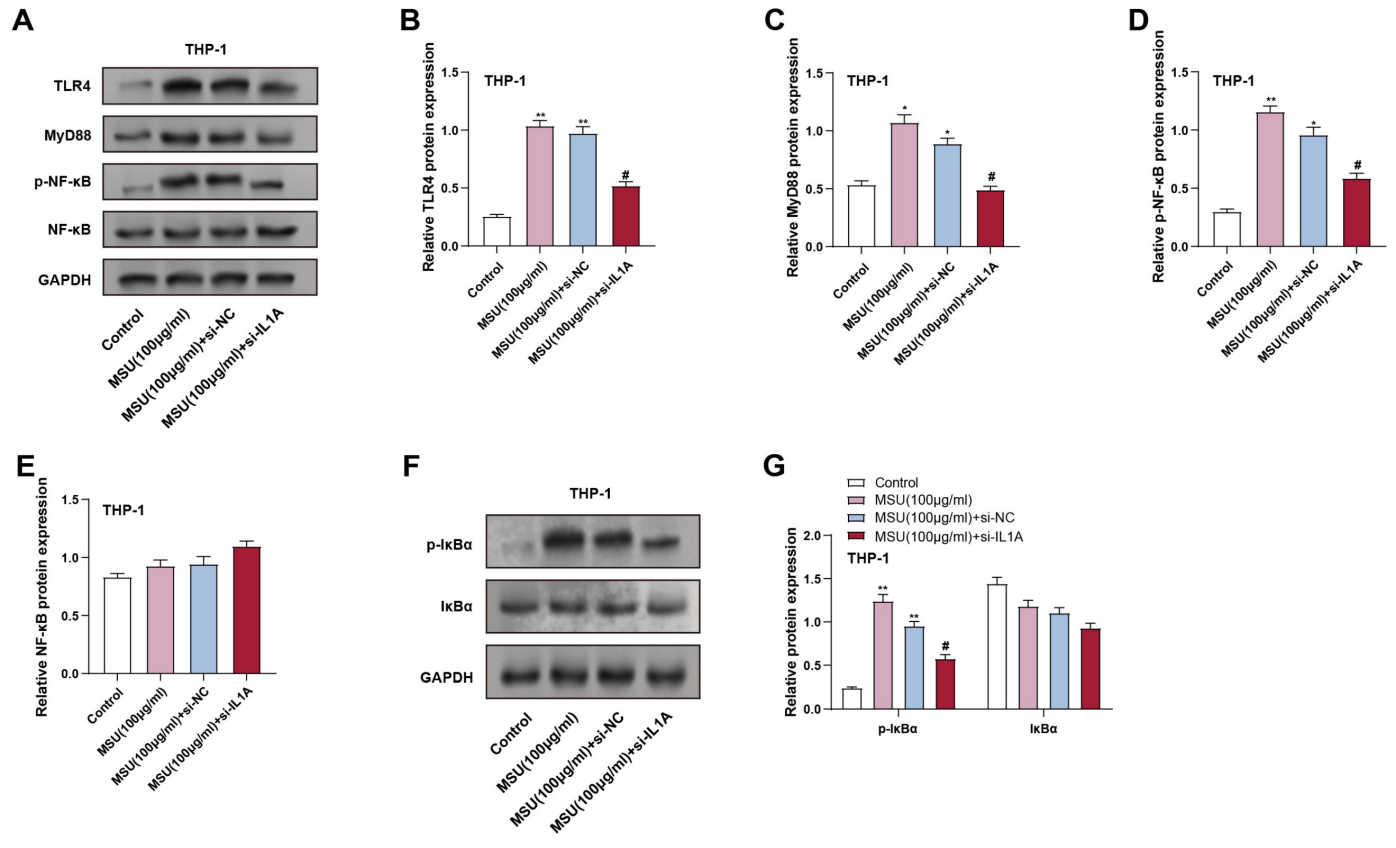
** Effect of *IL1A* knockdown on MSU-induced TLR4/MyD88/NF-κB signaling pathway activation in THP-1 macrophages.** (A) WB analysis of protein expression of TLR4, MyD88, p-NF-κB, and NF-κB in THP-1 cells treated under different conditions. (B-E) Quantification of TLR4 (B), MyD88 (C), p-NF-κB (D), and NF-κB (E) protein levels. ^**^P < 0.01 vs. Control; ^#^*P* < 0.05 vs. MSU (100 μg/ml) + si-NC. (F) WB analysis of protein expression of p-IκBα and IκBα in THP-1 cells treated under different conditions. (G) Quantification of p-IκBα (F) and IκBα (D) protein levels.^ **^P < 0.01 vs. Control; ^#^*P* < 0.05 vs. MSU (100 μg/ml) + si-NC. NC, negative control; MSU, monosodium urate.

The inflammatory response in macrophages is transmitted through the TLR4/MyD88/NF-κB pathway, which activates downstream pro-inflammatory genes. To investigate the result of* IL1A* knockdown on this pathway, we analyzed the expression of TLR4/MyD88-related proteins in THP-1 macrophages treated with MSU and *IL1A* knockdown. THP-1 macrophages were treated with 100 μg/mL MSU for 24 hours. Protein analysis indicated that treatment with MSU markedly elevated the expression of MyD88, TLR4, and p-NF-κB, compared to the control group, indicating that the pathway was activated. Conversely, *IL1A* knockdown (MSU + si-*IL1A*) effectively alleviated these increases, as shown in Figures 5A-5D. In addition, in THP-1 cells, we continued to analyze the downstream NF-κB signaling-related proteins p-IκBα and IκBα. MSU treatment led to a significant upregulation of p-IκBα expression, indicating pathway activation, while* IL1A* knockdown significantly reduced the p-IκBα level induced by MSU treatment (Figures 5F and 5G). Notably, total NF-κB and IκBα proteins' expression remained unchanged across all conditions (Figures 5A and 5E). The expression of total IκBα protein did not substantially vary across the groups. These outcomes suggest that* IL1A* knockdown may inhibit MSU-induced activation of the TLR4/MyD88/NF-κB pathway in macrophages, potentially reducing inflammatory responses.

### 3.6 *IL1A* knockdown and TLR4 inhibition synergistically suppress MSU-induced TLR4/MyD88/NF-κB pathway in macrophages

**Figure 6:**
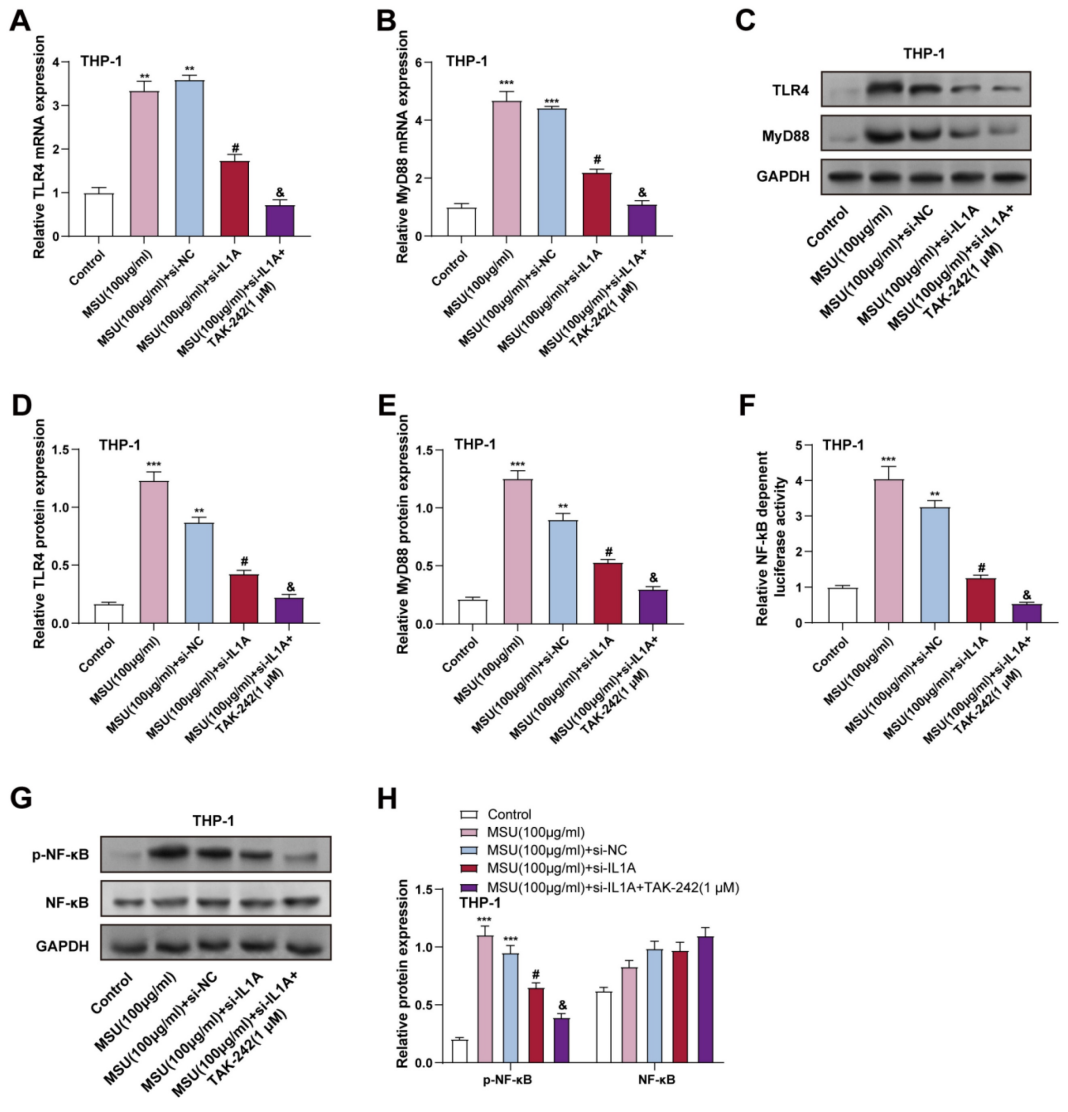
***IL1A* activates NF-κB through TLR4 and promotes NF-κB transcription.** (A and B) qRT-PCR was performed to analyze changes in* TLR4* (A) and *MyD88*(B) mRNA levels in THP-1 cells treated under different conditions. ^**^P < 0.0, ^***^P < 0.001 vs. Control; ^#^*P* < 0.05 vs. MSU (100 μg/ml) + si-NC; ^&^*P* < 0.05 vs. MSU (100 μg/ml) + si-*IL1A*. (C-E) WB analysis of TLR4 and MyD88 protein levels in THP-1 cells treated under different conditions. ^**^P < 0.01, ^***^P < 0.001 vs. Control; ^#^*P* < 0.05 vs. MSU (100 μg/ml) + si-NC; ^&^*P* < 0.05 vs. MSU (100 μg/ml) + si-*IL1A*. (F) The luciferase assay was used to measure the activity of NF-κB luciferase in treated THP-1 cells under different conditions. The Y-axis indicates the relative luciferase activity. ^**^P < 0.01, ^***^P < 0.001 vs. Control; ^#^*P* < 0.05 vs. MSU (100 μg/ml) + si-NC; ^&^*P* < 0.05 vs. MSU (100 μg/ml) + si-*IL1A*. (G and H) WB analyzed p-NF-κB and NF-κB protein expression in THP-1 cells treated under different conditions. ^**^P < 0.01, ^***^P < 0.001 vs. Control; ^#^*P* < 0.05 vs. MSU (100 μg/ml) + si-NC; ^&^*P* < 0.05 vs. MSU (100 μg/ml) + si-*IL1A*. qRT-PCR, quantitative real-time polymerase chain reaction; NC, negative control; MSU, monosodium urate.

To elucidate the role of *IL1A* in the activation of the TLR4/MyD88/NF-κB pathway, the result of *IL1A* knockdown on MSU-stimulated THP-1 cells was first analyzed. THP-1 cells were treated with 100 μg/mL MSU for 24 hours. WB analysis and qRT-PCR revealed that MSU treatment notably increased TLR4 and MyD88 expression, and in contrast to the MSU + si-NC group, *IL1A* knockdown (MSU + si-*IL1A*) significantly reduced the expression of TLR4 and MyD88, proving that *IL1A* may contribute to MSU-induced activation of this pathway (Figures 6A-6E). This indicates that *IL1A* contributes to MSU-induced activation of this pathway. Next, we investigated the effect of a selective TLR4 inhibitor (TAK-242) on the TLR4/MyD88/NF-κB pathway in *IL1A* knockdown. In the MSU + si-*IL1A* group, TAK-242 was then applied for 24 hours. TAK-242 treatment further inhibited the expression of TLR4 and MyD88, showing a synergistic inhibitory effect (Figures 6A-6E). The luciferase reporter gene experiment demonstrated that NF-κB transcriptional activity was elevated in MSU treatment and significantly reduced by *IL1A* knockdown. NF-κB transcriptional activity was significantly reduced after TAK-242 treatment, indicating effective inhibition of NF-κB activation (Figure 6F). In addition, WB analysis showed that the p-NF-κB level, which was elevated in MSU-treated cells, was significantly reduced in *IL1A* knockdown and further reduced in TAK-242 treatment. Total NF-κB protein expression remained unchanged across all conditions (Figures 6G and 6H). These findings suggest that *IL1A* knockdown alleviates MSU-induced activation of the TLR4/MyD88/NF-κB pathway in macrophages, and that TLR4 inhibition by TAK-242 amplifies this inhibitory effect, further reducing inflammatory signaling.

### 3.7 *IL1A* knockdown reduces proinflammatory cytokine expression and NLRP3 inflammasome activation in MSU-induced macrophages

**Figure 7:**
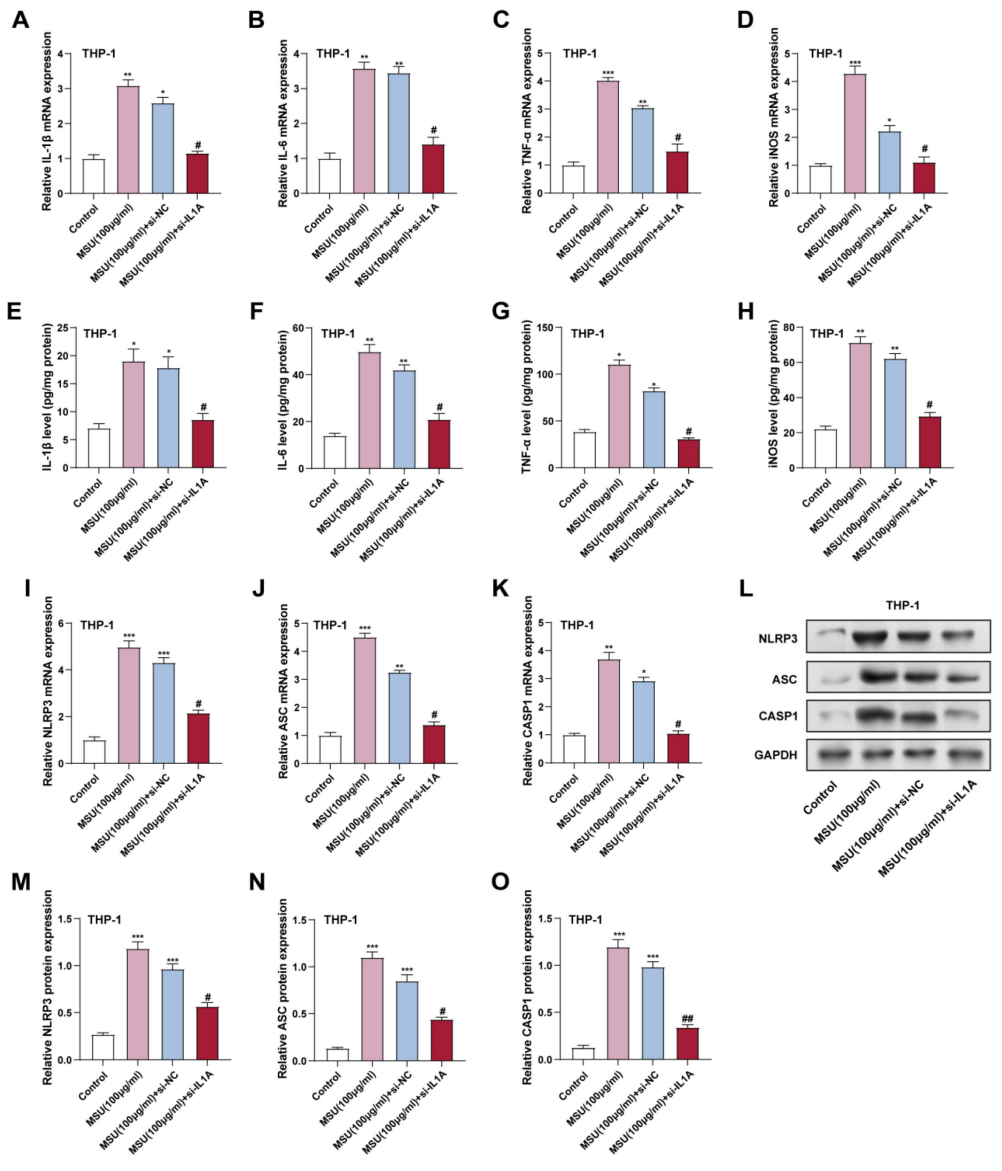
** Downregulation of *IL1A* reduces NF-κB-dependent inflammatory cytokine and inflammasome expression in MSU-treated THP-1 cells.** (A-D) qRT-PCR was performed to analyze the mRNA expression levels of NF-κB downstream inflammatory cytokines *IL-1β*, *IL-6*, *TNF-α*, and *iNOS* in THP-1 cells treated under different conditions. ^*^P < 0.05,^ **^P < 0.01, ^***^P < 0.001 vs. Control; ^#^P < 0.05 vs. MSU (100 μg/ml) + si-NC. (C-H) ELISA was performed to detect IL-1β, IL-6, TNF-α, and iNOS protein levels in THP-1 cells under different conditions. ^*^P < 0.05, ^**^P < 0.01 vs. Control; ^#^P < 0.05 vs. MSU (100 μg/ml) + si-NC. (I-K) qRT-PCR was performed to analyze the mRNA expression levels of *NLRP3* (I), *ASC* (J), and *CASP1* (K) in THP-1 cells treated under different conditions. ^*^P < 0.05, ^**^P < 0.01, ^***^P < 0.001 vs. Control; ^#^*P* < 0.05 vs. MSU (100 μg/ml) + si-NC. (L) WB analysis of the expression levels of NLRP3, ASC, and CASP1 proteins in THP-1 cells under different conditions. (M-O) Quantification of NLRP3 (M), ASC (N), and CASP1 (O) protein expression levels. ^***^P < 0.001 vs. Control; ^#^*P* < 0.05, ^##^*P* < 0.01 vs. MSU (100 μg/ml) + si-NC. qRT-PCR, quantitative real-time polymerase chain reaction; NC, negative control; MSU, monosodium urate; ELISA, enzyme-linked immunosorbent assay.

To evaluate the effect of *IL1A* knockdown on MSU-induced inflammatory responses in THP-1 macrophages, we assessed the expression of key proinflammatory cytokines and NLRP3 inflammasome components at the mRNA and protein levels. THP-1 macrophages were treated with 100 μg/mL MSU for 24 hours. qRT-PCR experiments analyzed TNF-α, iNOS, IL-1β, and IL-6 mRNA levels in THP-1 cells. MSU treatment (100 µg/mL), inflammatory factors mRNA levels were markedly elevated compared to the control group (Figure 7A-7D). However, *IL1A* knockdown (MSU + si-*IL1A*) significantly reduced these mRNA levels relative to the MSU + si-NC group, indicating that *IL1A* contributes to the MSU-induced expression of these cytokines. Consistent with the mRNA results, in THP-1 cells, ELISA analysis indicated that MSU treatment significantly raised the protein levels of inflammatory factors. In contrast, *IL1A* knockdown in the presence of MSU (MSU + si-*IL1A*) resulted in a notable decline in these protein levels, contrasted with the MSU + si-NC group, with IL-1β levels decreasing by 45%, IL-6 levels decreasing by 38%, and TNF-α levels decreasing by 40% relative to MSU treatment alone (Figures 7E-7H). These findings suggest that *IL1A* knockdown can decrease MSU-induced macrophage cytokine production. The effects of *IL1A* suppression on the inflammasome NLRP3, a key mediator of macrophage inflammation, were investigated further. NLRP3, ASC, and CASP1 mRNA expression levels were considerably elevated after MSU treatment, as shown by qRT-PCR (Figures 7I-7K). *IL1A* knockdown (MSU + si-*IL1A*) dramatically decreased the mRNA levels of these inflammasome components when compared to MSU + si-NC, suggesting that *IL1A* plays a role in the elevation of NLRP3 inflammasome genes in response to MSU. The results of the protein-level investigation also supported this. Given that it reduces MSU-induced proinflammatory cytokine production and NLRP3 inflammasome activation in THP-1 macrophages, our results imply that *IL1A* silencing may have an anti-inflammatory effect on macrophage-mediated inflammatory responses.

## 4. Discussion

The pathophysiology of gout, an inflammatory arthritis brought on by MSU crystal deposition, is significantly influenced by the proinflammatory cytokine* IL1A*^28^*.* Studies by Ling *et al.* have shown that *IL1A* regulates gout inflammation primarily through the TLR signaling pathway, and its elevated expression in gout patients makes it a potential diagnostic marker^25^. The role of *IL1A* in promoting inflammatory responses is vital as it directly contributes to the progression of gout. In addition, studies by Gross *et al.* highlighted that* IL1A* is released during gout inflammasome activation through both inflammasome-dependent and -independent mechanisms, emphasizing its involvement in inflammatory pathways^29^. Our study observed that MSU treatment significantly increased *IL1A* expression in HUVECs and THP-1 cells, with a dose-dependent increase at 50 μg/mL instead of 100 μg/mL. *IL1A* knockdown effectively reduced MSU-induced upregulation in both cell types, alleviated MSU-induced apoptosis, and inhibited cell growth in HUVECs. These findings reinforce the function of* IL1A* in regulating gout inflammation and cell damage. In addition, Colantuoni *et al.* proposed that IL-1RA produced by modified immune cells can counteract IL-1-mediated inflammation, offering a potential method of treating gout and other conditions caused by IL-1^22^.

Because endothelial cells are subject to MSU crystals in gouty arthritis, which causes local inflammation and vascular involvement, HUVECs are frequently employed to research endothelial function and inflammation^30^. HUVECs might not, however, adequately represent the intricate immune response seen in gout. Since macrophages are essential to the immune response and major contributors to MSU-induced inflammation, combining them with a macrophage cell line, such as THP-1 cells, offers a more thorough method to replicate the inflammatory milieu of gout more accurately^31^. Utilizing THP-1 macrophages and HUVECs enables a more representative investigation of the inflammatory processes and cellular connections linked to gout. By reacting to MSU crystals, Liu L *et al.* showed that macrophages are essential in acute gout^32^. A more thorough examination of the interactions between endothelial and immune cells is made possible by co-culturing HUVEC and MSU-treated THP-1 cells, which sheds light on the inflammatory processes associated with gout. MSU and calcium pyrophosphate (CPP) crystals activate macrophages through GLUT1-mediated glycolysis, which triggers NLRP3 and IL-1β activation, hence increasing inflammation in gout and pseudo-gout^33^. This change in metabolism identifies possible treatment targets for illnesses brought on by crystals. Furthermore, Li *et al.* discovered that Kv1.5 plays a regulatory function in the electrical remodeling of atrial myocytes when MSU-induced activation of the NLRP3 inflammasome in macrophages occurs^34^. Furthermore, our co-culture experiment demonstrated that knocking down *IL1A* in THP-1 macrophages protected HUVECs from MSU-induced cell death, as evidenced by increased cell viability and altered expression of apoptosis-related proteins, including higher Bcl-2 and lower cleaved caspase-3 levels.

The NLRP3 inflammasome and the TLR4/MyD88/NF-κB pathway are essential to controlling inflammation and immunological responses, especially in gout^24^. PAMPs are acknowledged by TLR4, which then triggers downstream signaling via the adaptor protein MyD88 and NF-κB activation^35^. A crucial transcription factor called NF-κB controls genes linked to pro-inflammatory mediators, all of which increase inflammatory reactions^36^. Additionally, inflammasomes, such as NLRP3, ASC, and CASP1, respond to cellular stress by activating caspase-1, IL-1β is broken down into its active form, further intensifying inflammation^37^. In gout, NF-κB and NLRP3 signaling interactions contribute significantly to tissue damage and inflammation. Shen *et al.* reported that luteolin exerts anti-inflammatory effects in gouty arthritis by downregulating TLR4/MyD88/NF-κB signaling, reducing cytokine levels, and joint swelling^38^. Similarly, *Dioscorea collettii* extract attenuates MSU-induced inflammation by preventing the TLR4/MyD88/NF-κB pathway^39^, and caffeic acid phenethyl ester (CAPE) suppresses NLRP3 activation by disrupting NLRP3-ASC interaction^40^.

In our study, *IL1A* knockdown effectively attenuated MSU-induced activation of the TLR4/MyD88/NF-κB pathway and the NLRP3 inflammasome in THP-1 macrophages. Specifically, *IL1A* downregulation reduced TLR4, MyD88, and phosphorylated NF-κB expression, demonstrating that *IL1A* contributes to the pro-inflammatory signaling cascade in response to MSU. Furthermore, IL-1α signals through the IL-1 receptor type I (IL-1RI), which utilizes the same adaptor protein MyD88 as TLR4, leading to NF-κB activation. In the setting of MSU stimulation, IL-1α released from macrophages may act in an autocrine or paracrine manner to activate NF-κB via IL-1RI. This sustained NF-κB activation could enhance TLR4 expression or sensitivity, thereby establishing a positive feedback loop that amplifies inflammatory signaling. Accordingly, *IL1A* knockdown may attenuate this feedback mechanism, reducing TLR4/MyD88/NF-κB pathway activation. This mechanistic hypothesis is supported by our observation that *IL1A* silencing downregulated TLR4 and MyD88 protein levels and synergized with TAK-242, a TLR4 inhibitor, to suppress NF-κB activity further. These findings suggest that IL-1α initiates inflammatory signaling via IL-1RI and may reinforce TLR4-driven responses, contributing to persistent inflammation in MSU-induced conditions. Moreover, *IL1A* knockdown reduced MSU-induced increases in pro-inflammatory cytokines and inflammasome components (NLRP3, ASC, CASP1), as shown by qRT-PCR and ELISA. This indicates that *IL1A* is essential for coordinating NF-κB and inflammasome activation, exacerbating inflammatory cytokine production and cellular apoptosis.

This study highlights a novel mechanistic role for IL-1α in amplifying TLR4/MyD88/NF-κB signaling and inflammasome activation in MSU-stimulated macrophages. By demonstrating that *IL1A* knockdown attenuates pro-inflammatory signaling and protects endothelial cells in a co-culture model, we provide new insights into how IL-1α may sustain gout-associated inflammation through intercellular communication. However, we acknowledge that these findings are based on *in vitro* models using THP-1-derived macrophages and HUVECs, which, although mechanistically informative, may not fully recapitulate the complex cellular interactions and systemic factors present in gouty arthritis *in vivo*. The use of conditioned media and dual cell types partially addresses this limitation. *In vivo* validation in gout animal models or clinical patient samples will be essential to confirm the translational potential of targeting IL-1α. From a translational perspective, IL-1 blockade has already shown clinical benefit in gout management. For instance, anakinra, a recombinant IL-1 receptor antagonist (IL-1Ra), has been used to treat refractory gout flares, particularly in patients who cannot tolerate traditional therapies. Our findings complement this strategy by suggesting that targeting IL-1α specifically may further fine-tune inflammatory control. Notably, the work by Colantuoni *et al.* highlights the potential of IL-1 targeting approaches, reinforcing the relevance of our findings to clinical intervention in gout^22^. Future studies could explore IL-1α neutralization in animal models of gout to evaluate the therapeutic potential suggested by our *in vitro* findings.

## 5. Conclusion

Our results have significant ramifications for the pathophysiology of gout, as it is a crucial regulator of MSU-induced inflammation and apoptosis. *IL1A* knockdown showed an anti-inflammatory impact by reducing pro-inflammatory cytokine levels (IL-1β, IL-6, TNF-α, iNOS), MSU-induced apoptosis in HUVECs, and NLRP3 inflammasome activation in THP-1 macrophages. Furthermore, the TLR4 inhibitor TAK-242 enhanced the TLR4/MyD88/NF-κB signaling pathway, which was decreased by *IL1A* knockdown. These results imply that *IL1A* modulates the relationship between the inflammasome and NF-κB pathways in gout-related inflammation. Targeting *IL1A*, either alone or in combination with TLR4 suppression, may be a promising treatment strategy for gout-related inflammation and tissue damage.

## Supplementary Material

Supplementary figure.

## Figures and Tables

**Table 1:** Primer sequences for qRT-PCR.

Target	Direction	Sequence (5'-3')
*IL1A*	Forward	CTTGTCCCTCCCTGGTTTGAA
*IL1A*	Reverse	ACAGATTGATCCATGCAGCCT
TLR4	Forward	CTCCTGCGTGAGACCAGAAA
TLR4	Reverse	CTCCGTGATAAAACGGCAGC
MyD88	Forward	ACTTGGAGATCCGGCAACTG
MyD88	Reverse	ATCCGGCGGCACCAATG
IL-1β	Forward	CAGAAGTACCTGAGCTCGCC
IL-1β	Reverse	GGTCGGAGATTCGTAGCTGG
IL-6	Forward	TTCGGTCCAGTTGCCTTCTC
IL-6	Reverse	CTGAGATGCCGTCGAGGATG
TNF-α	Forward	CACAGTGAAGTGCTGGCAAC
TNF-α	Reverse	AGGAAGGCCTAAGGTCCACT
iNOS	Forward	CCCCGGCCTTCTGTTTACAT
iNOS	Reverse	TCAAGTGAGGCCTGGATTCG
NLRP3	Forward	GCTGGCATCTGGGGAAACCT
NLRP3	Reverse	CAAGTCCACATCCTCCAGGTC
ASC	Forward	ATCCAGGCCCCTCCTCAG
ASC	Reverse	AGAGCTTCCGCATCTTGCTT
CASP1	Forward	ATCCGTTCCATGGGTGAAGG
CASP1	Reverse	GCCCCTTTCGGAATAACGGA
GAPDH	Forward	AATGGGCAGCCGTTAGGAAA
GAPDH	Reverse	GCGCCCAATACGACCAAATC
