# Supplementary figures and images for: IL1A regulates MSU-induced apoptosis and inflammatory response through TLR4/MyD88/NF-κB signaling pathway
Source: Int J Med Sci. 2025 Jun 23;22(12):3070–83. doi: 10.7150/ijms.112102 (PMC12244086; doi:10.7150/ijms.112102)

## Supplementary Figure 1

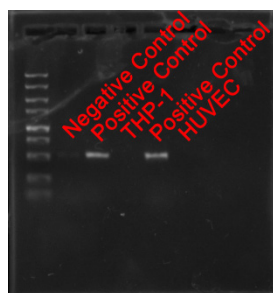

Supplement: Supplementary file 1 — Supplementary figure. [file ijmsv22p3070s1.pdf]
